# Supplementary material for: Human single-neuron activity is modulated by intracranial theta burst stimulation of the basolateral amygdala
Source: bioRxiv. 2025 Feb 15:2024.11.11.622161. Originally published 2024 Nov 12. Preprint. [Version 2] doi: 10.1101/2024.11.11.622161 (PMC11601271; doi:10.1101/2024.11.11.622161)
Supplement: Supplementary file 1 [file NIHPP2024.11.11.622161v2-supplement-1.pdf]

bioRxiv preprint doi: https://doi.org/10.1101/2024.11.11.622161; this version posted February 15, 2025. The copyright holder for this preprint (which was not certified by peer review) is the author/funder, who has granted bioRxiv a license to display the preprint in perpetuity. It is made available under aCC-BY 4.0 International license.

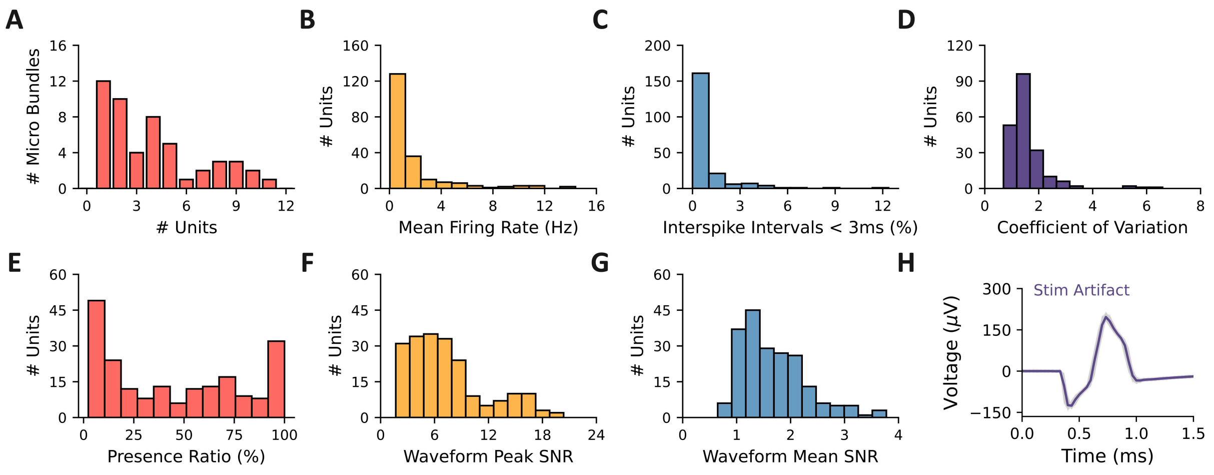

Supplemental Figure 1: Unit quality metrics.

(A) Number of units detected per implanted microelectrode bundle. (B) Mean firing rate (Hz) across recording session. (C) Percent of interspike intervals < 3 ms. (D) Interspike interval coefficient of variation. (E) Mean presence ratio of firing within units (1 s bins). (F) Signal-to-noise ratio of unit waveform peak. (G) Mean signal-to-noise ratio across the entire unit waveform. (H) Representative example of stereotyped, high-amplitude stimulation-artifact waveform; non-physiological waveforms were excluded from analysis.

bioRxiv preprint doi: https://doi.org/10.1101/2024.11.11.622161; this version posted February 15, 2025. The copyright holder for this preprint (which was not certified by peer review) is the author/funder, who has granted bioRxiv a license to display the preprint in perpetuity. It is made available under aCC-BY 4.0 International license.

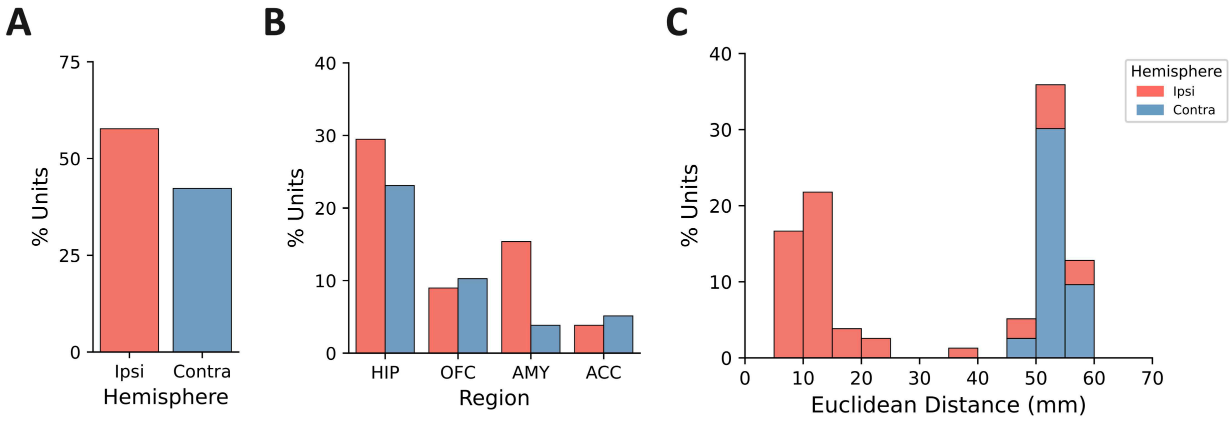

Supplemental Figure 2: Characterization of units based on laterality relative to stimulation.

(A) Unit counts on the contralateral (Contra) or ipsilateral (Ipsi) side of stimulation. (B) Unit counts separated by laterality and region. (C) Stacked histograms of Euclidean distance between microelectrode bundle location and stimulation contacts, separated by laterality (bin size = 5 mm).

bioRxiv preprint doi: https://doi.org/10.1101/2024.11.11.622161; this version posted February 15, 2025. The copyright holder for this preprint (which was not certified by peer review) is the author/funder, who has granted bioRxiv a license to display the preprint in perpetuity. It is made available under aCC-BY 4.0 International license.

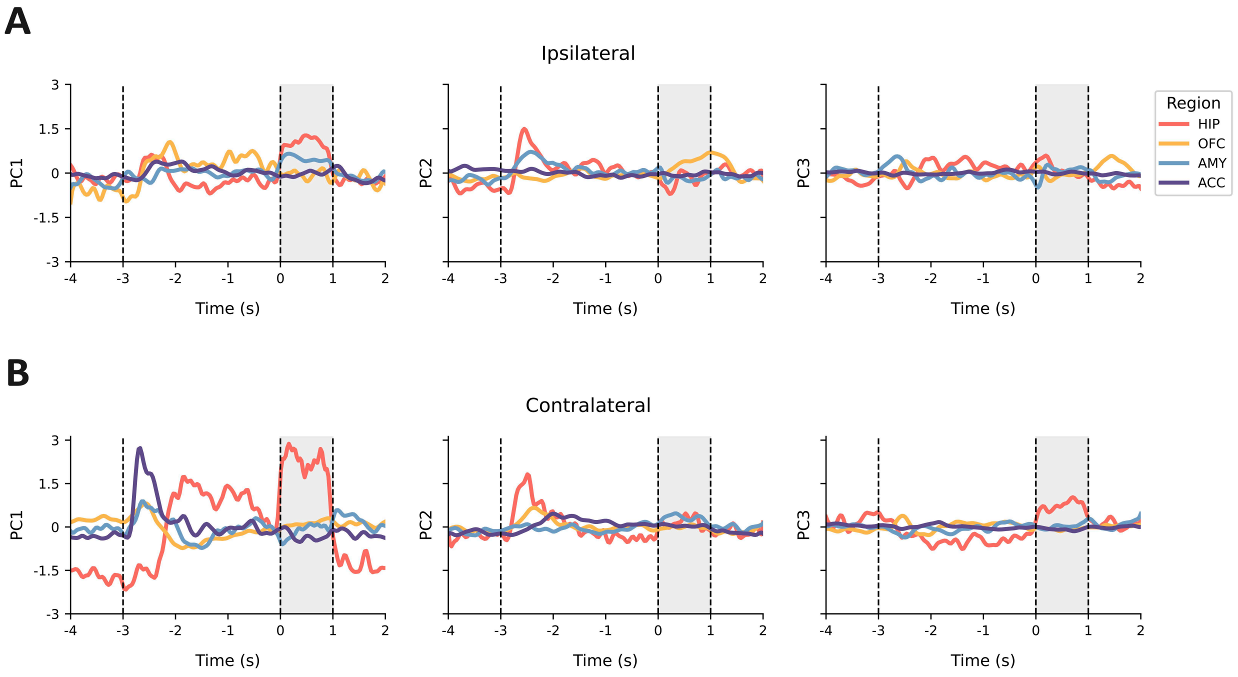

Supplemental Figure 3: Analysis of pseudo-population activity within regions, separated by laterality relative to stimulation.

Pseudo-population activity was characterized within each region via a linear dimensionality reduction on the trial-averaged firing rates. The temporal dynamics of each region's first three principal components are shown for (A) units ipsilateral to stimulation and (B) units contralateral to stimulation. HIP = hippocampus (coral), OFC = orbitofrontal cortex (yellow), AMY = amygdala (blue), ACC = anterior cingulate cortex (purple).

bioRxiv preprint doi: https://doi.org/10.1101/2024.11.11.622161; this version posted February 15, 2025. The copyright holder for this preprint (which was not certified by peer review) is the author/funder, who has granted bioRxiv a license to display the preprint in perpetuity. It is made available under aCC-BY 4.0 International license.

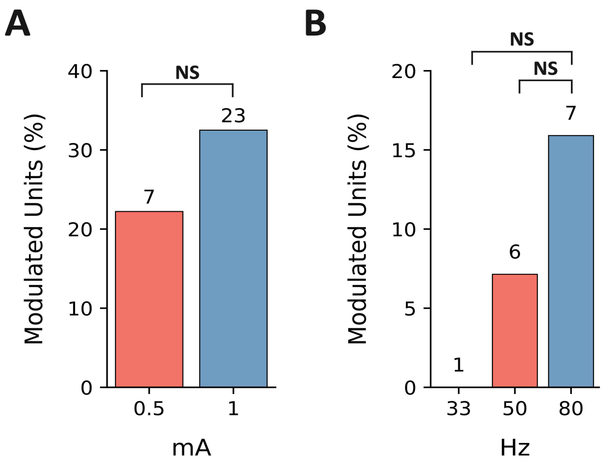

Supplemental Figure 4: Sub-analysis of stimulation parameters used across experiments.

Comparison of the proportion of stimulation-modulated units across sessions with 1 mA (n = 23) vs. 0.5 mA (n = 7). (B) Comparison of the proportion of stimulation-modulated units across sessions testing distinct pulse frequencies: 33 Hz vs. 80 Hz (n = 1) and 50 Hz vs. 80 Hz (n = 6). The values above individual bars represent the number of sessions using that stimulation parameter. NS = not significant.

bioRxiv preprint doi: https://doi.org/10.1101/2024.11.11.622161; this version posted February 15, 2025. The copyright holder for this preprint (which was not certified by peer review) is the author/funder, who has granted bioRxiv a license to display the preprint in perpetuity. It is made available under aCC-BY 4.0 International license.

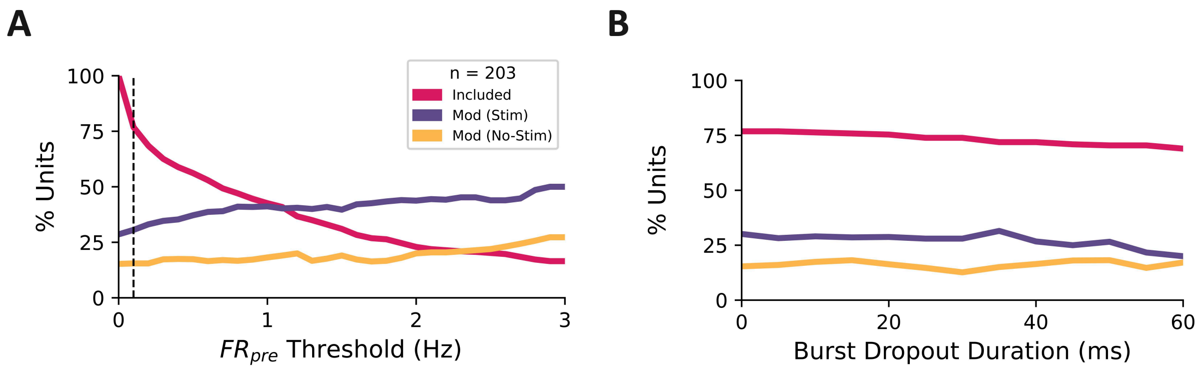

Supplemental Figure 5: Control analyses for the detection of modulated units. The same permutation-based analyses reported in the manuscript were repeated under different control conditions. The percent of units (total n = 203) that met the firing rate threshold for inclusion (pink), the percent of included units modulated in the stim condition (purple), and the percent of included units modulated in the no-stim condition are shown. (A) The threshold for inclusion of units was varied from 0–3 Hz (0.1 step size); the black dashed line represents the ≥ 0.1 Hz threshold used in the manuscript. (B) To control for the possibility that non-physiological stimulation artifacts may preclude the detection of temporally adjacent spiking, we removed segments of data beginning at the onset of each burst of pulses (0–60 ms, 5 ms step size). Identical temporal windows were removed from the corresponding pre- and post-stimulation epochs to mitigate effects resulting solely from summation over different epoch sizes (reduced spike counts with shorter windows).
